# Annotation matters: validating the discovery of cancer drivers

**DOI:** 10.1080/23723556.2020.1806679

**Published:** 2020-08-31

**Authors:** Marta Rodríguez-Martínez, Jesper Q. Svejstrup

**Affiliations:** Mechanisms of Transcription Laboratory, The Francis Crick Institute, London, UK

**Keywords:** STK19, annotation, cancer driver, kinase

## Abstract

Advanced sequencing techniques have helped unveil numerous new, potential cancer driver mutations. However, manual curation and analysis of gene and protein annotation are essential to verify such discoveries. Our recent study of *STK19* (Serine Threonine Kinase 19), a previously identified melanoma driver, is a clear example of the importance of such detailed analysis, with both STK19 gene and protein annotations in frequently used databases having been proven incorrect.

In the past few years, targeted sequencing as well as whole genome- or exome-sequencing have allowed researchers to explore the alterations in cancer genomes, with thousands of cancer genomes having been analyzed to allow the identification of new cancer driver genes and mutations. However, the methods for uncovering cancer drivers rely on the accurate annotation of the human genome, which is often imperfect and can therefore lead to misinterpretation of the results. An illustrative example of this imperfection is found in the recent comparison of the RefSeq and Gencode human gene databases, where only 27.5% of transcripts annotated in Gencode are shared by the RefSeq annotation.^1^

The gene encoding what is now known as Serine Threonine Kinase 19 (STK19) provides a further illustrative example of such misinterpretation.^2^ Indeed, the identified melanoma driver mutation STK19 D89N^3^ turned out not to be in the coding region of the expressed form of STK19, which in reality lacks the first 110 amino acids shown in database annotations. The mutation is instead non-coding and positioned near the *STK19* transcription start site (TSS), but does not affect gene or protein expression either.^2^ It thus cannot be a cancer driver.

Cutaneous melanoma is the most aggressive form of skin cancer. It typically develops on sun-exposed skin and is linked with DNA damage arising from UV-irradiation. These melanomas have one of the highest tumor mutation burdens of all malignancies, with the alterations typically comprising UV-induced C > T transitions occurring at di-pyrimidines.^3,4^ The high background tumor mutation burden makes it challenging to identify significantly mutated genes that drive this cancer. Alkallas and coworkers recently published a mutation significance study of over 1,000 melanoma exomes, which they combined with multi-omic analysis of 470 cases from the Cancer Genome Atlas.^5^ This approach allowed them to not only identify new significantly mutated genes with co-occurring loss-of-heterozygosity and loss-of-function mutations, but also to rule out a number of false positives, identified based on the proximity of their mutation to the coding area of an unexpressed gene isoform. Not surprisingly, STK19 was identified as one of these false positives.^5^

Moreover, recent studies have shown that binding of the transcription factor ETS (ETS1/ETS2) can reduce DNA repair efficiency, resulting in the accumulation of specific mutation signatures near these sites and thus the TSS of certain genes.^6,7^ The mutations caused by ETS binding, which almost certainly include the misannotated *STK19* D89N driver mutation, ^2^ can be induced by simple UV-irradiation of cells, and they typically have no effect on gene or protein expression, ^8^ corroborating the lack of association with malignant transformation. Intriguingly, mutations in the human *TERT* (Telomerase Reverse Transcriptase) promoter have been found in benign skin nevi, arguing against a role of these well-known mutations in cancer progression, and suggesting that further analysis is required before extending the use of this mutation as a biomarker for cutaneous melanoma.^9^

Unfortunately, in the case of STK19, a second potential misconception was faced due to the old annotation of the protein as a serine threonine kinase.^10^ The previous kinase activity studies were typically done using protein pull-downs from insect cells^10,11^ and therefore the observed activity could well have been due to contamination, or to an STK19-interacting kinase rather than STK19 itself. Indeed, in spite of repeated attempts, we have been unable to detect STK19 kinase activity, ^2^ and no evidence of STK19 kinase activity using highly purified protein has ever been reported. Equally damningly, the STK19 protein has none of the domain- or sequence features expected of a protein kinase. Indeed, in their response to our recent study uncovering the misconceptions about STK19, ^2^ the authors of the previous study that identified STK19 as an NRAS kinase with a role in cancer progression, ^11^ now acknowledge that STK19 is unlikely to have intrinsic kinase activity.^12, 13^

In summary, although STK19 represents an unusual case in which both protein function and gene expression characteristics have been misannotated (Figure 1), it represents proof in point that such mistakes do occur, and that careful manual analysis of basic gene and protein characteristics should be carried out once potential cancer drivers have been identified by sophisticated algorithms. In the case of STK19, basic misconceptions unfortunately led the field to believe that STK19 represents a great opportunity to develop targeted therapies for aggressive melanoma, with two separate studies reporting progress in producing small molecule inhibitors.^11,13^ Our analysis thus also underscores the importance of carefully analyzing newly discovered cancer drivers before resources are dedicated to therapy development.

**Figure 1.**
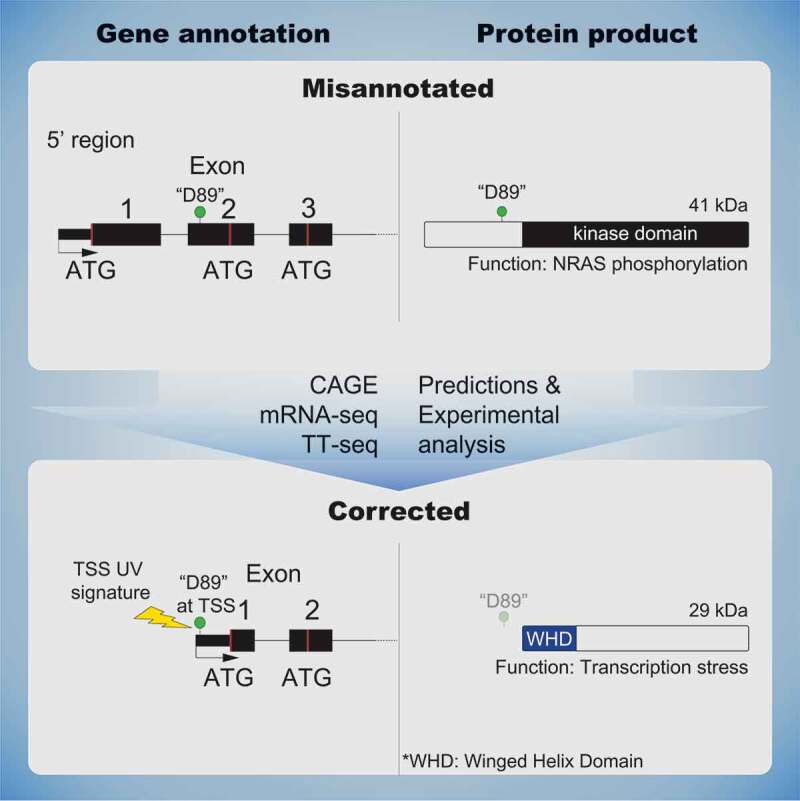
Schematic representation of the current (misannotated) and corrected STK19 gene and protein annotations. ATGs are indicated by red lines and the position of the (non-existent) D89 by a green pin. For gene annotation (left panel), only the 5ʹ region of the gene is shown, as this is where the annotation correction takes place. In the right panel, depicting protein annotation, the respective sizes of the encoded protein, as well as the proposed protein function, is indicated next to the diagram. The central arrow indicates the methods and tools used in^2^ to correct the current gene and protein annotation
